# Humidity influenced capacitance and resistance of an Al/DNA/Al Schottky diode irradiated by alpha particles

**DOI:** 10.1038/srep25519

**Published:** 2016-05-10

**Authors:** Hassan Maktuff Jaber Al-Ta’ii, Yusoff Mohd Amin, Vengadesh Periasamy

**Affiliations:** 1 Low Dimensional Materials Research Centre (LDMRC), Department of Physics, Faculty of Science, University of Malaya, 50603 Kuala Lumpur, Malaysia; 2 Department of Physics, Faculty of Science, University of Al-Muthanna, 66001, Iraq; 3 Department of Physics, Faculty of Science, University of Malaya, 50603 Kuala Lumpur, Malaysia

## Abstract

Deoxyribonucleic acid or DNA based sensors, especially as humidity and alpha particle sensors have become quite popular in recent times due to flexible and highly optimizable nature of this fundamental biomaterial. Application of DNA electronics allow for more sensitive, accurate and effective sensors to be developed and fabricated. In this work, we examined the effect of different humidity conditions on the capacitive and resistive response of Aluminum (Al)/DNA/Al Schottky barrier structure when bombarded by time-dependent dosages of alpha particles. Based on current-voltage profiles, which demonstrated rectifying behaviours, Schottky diode parameters such as ideality factor, barrier height and series resistance was calculated. Results observed generally pointed towards a decrease in the resistance value from the pristine to the radiated structures. It was also demonstrated that under the effect of humidity, the capacitance of the DNA thin film increased from 0.05894 to 92.736 nF, with rising relative humidity level. We also observed the occurrence of the hypersensitivity phenomena after alpha irradiation between 2 to 4 min by observing a drop in the series resistance, crucial in the study of DNA damage and repair mechanisms. These observations may also suggest the exciting possibility of utilizing Al/DNA/Al Schottky diodes as potentially sensitive humidity sensors.

The quantity of water vapor in the atmosphere is called humidity, which affects all environmental biological and chemical processes. Humidity also negatively affects various industrial-manufacturing methods if proper steps are not taken. As such, humidity level in terms of relative humidity (RH) are closely monitored to reveal fluctuations in situations ranging from high to low temperatures or in mixtures with other gases^1^. RH equals to the ratio between the quantity of wetness or content of air to the extreme (saturated) moisture level that the air can hold at a given pressure and temperature^2^. Therefore, RH values are recorded and studied extensively as it has many applications that includes improving indoor air quality for comfortable and healthy living conditions^3^.

Flexible, chipless and wireless humidity sensors are built based on the difference in dielectric constant (capacitive), proton/ionic conduction (resistive), refractive index (optical), frequency (impedance) or mass of the active material against humidity level^4^. Drop casting and spin coating are simple methods that can be used to sediment the active organic semiconductor at low temperatures. These effective processes may be applied in commercial organic semiconductor technology as an alternative to the conventional inorganic semiconductor detecting materials^5^. Generally, humidity sensors can be divided into resistive, thermo elemental capacitive, oscillating, and mechanical types using sensitive organic materials^6^,^7^. There are several factors that act to determine the performance and the advantage of the humidity-measuring instrument. These include properties such as fast reaction time, physically and chemically stable active compounds, linear behavior, suitable detection range, good strength, resistance against pollution and cheaper fabrication cost^8^,^9^,^10^,^11^. In this context, deoxyribonucleic acid (DNA) has been reported to demonstrate properties useful for utilization as a humidity sensing material. For example, double-stranded DNA (dsDNA) structure is temperature stable up to 100 °C with low optical loss over a broad wavelength domain^12^.

Han *et al.* and Otsuka *et al.* observed the RH value effect on the conductance of samples involving various DNA molecules. They reported that poly(dC)-poly(dG) sample demonstrated a higher sensitivity to RH^13^,^14^. The activation energy was also observed to be lower than the poly(dT)-poly(dA) sample^14^. Tabata *et al.*^15^ meanwhile reported that the resistance of DNA film declined progressively with increasing moisture from 35 to 70%. The dramatic resistance changes correspond to the variation in the charge conduction mechanism, which changes from electrical to ionic conduction^15^. Tuukkanen *et al.* observed that the conductivity of DNA of length 140 nm demonstrated insulating behaviors when placed in dry environment (about 30% RH). This was in contrast to improved conductivity under moist environment (80–90% RH)^16^. In other works, Kleine-Ostmann *et al.* and Yamahata *et al.* observed and reported that the conductivity followed the increase in humidity level exponentially in dsDNA and single-strand DNA (ssDNA) due to the absorption of water molecules to the nanostructured film surface^17^,^18^. Yamada and Sugiyama observed the response of bio-matrices consisting of metals such as Co^+2^ and Ni^+2^ mixed with DNA. A color transition from blue to red with change in the UV absorption due to the humidity effect was observed. This type of sensors are flexible, non-hazardous and cheap to fabricate^19^. In another work, Paul *et al.* used DNA functionalized carbon nanotube (DFC) networks to develop a field effect transistor (FET)-based humidity sensor. They reported that the charge transfer mechanism between the DNA and the water molecules caused the exponential variation of conductance of the DFC network with RH^20^.

Structures of DNA are strongly influenced by the environmental conditions due to their polymorphic nature^21^,^22^, which may be utilized in various exciting applications^23^. In industry, the utilization of metal-oxide semiconductors^24^, conventional semiconductors^25^,^26^ and organic semiconductors^27^,^28^ have led to fabrication of high-speed Schottky diodes. Taniguchi *et al.* observed that the ionic conduction through water layers under atmospheric conditions was the dominant conduction mechanism by using the frequency (100-1 KHz). Utilizing micro- and nanoelectrodes of DNA film, they observed that the semicircle becomes smaller in the high frequency range at high humidity^29^. In another work, Bi *et al.* demonstrated that the sensitivity of Graphene Oxide (GO) at 1 KHz was higher than that at 100 Hz and 10 KHz of frequencies^30^. Chen *et al.* meanwhile found that for carbon film, the capacitive response at 1 kHz was over 200% for RH shifting from 11 to 95%, compared with other frequencies^31^. In our work, we chose the mid-frequency at 0.8 KHz since capacitance decreased with increase in frequency corresponding to the operating parameters of the LCR meter used.

Generally, the experiments involved measuring various diode parameters against bombardment of alpha particles under various humidity conditions. As such, the data provides information of humidity dependent electronic parameters of sensitive DNA sensing material. The results, as clearly observed shows a distinct behavior specific to the humidity level used. These features, we believe, provides an effective avenue towards accurately and effectively sensing humidity fluctuations using the DNA molecules, which are totally biodegradable and abundant in nature besides having a high degree of flexibility. It is also very well known that humidity highly influences the semiconducting behavior of DNA, forming a solid reason behind the motivation into utilizing DNAs as a humidity sensing organic material. The experiments conducted in these studies have also brought to light the possibility of interrogating various important DNA related processes especially damage and repair mechanisms based on the occurrence of the interesting effect of hypersensitivity.

In this current study, we developed and utilized for the first time, a novel Al/DNA/Al surface-type Schottky diode as the humidity sensor. The fabricated Schottky diodes are further studied by investigating the fundamental changes induced by different time-dependent dosages of alpha particle irradiation on the DNA molecules. This was achieved by measuring its electrical characteristics under controlled chamber humidity to establish its humidity sensing potentials.

## Results and Discussion

**Figure 1:**
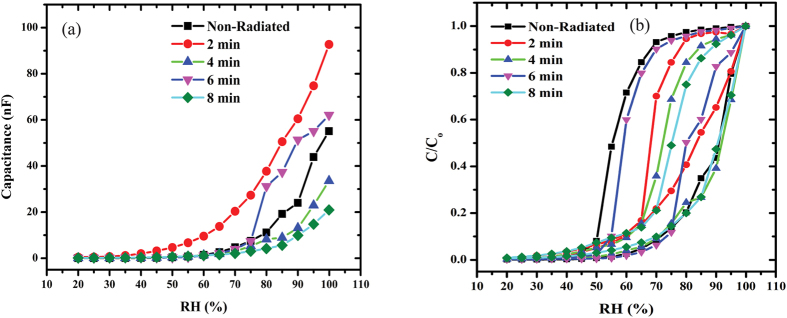
Capacitance versus relative humidity for the Al/DNA/Al humidity sensor.

Fig. 1(a) shows the relationship between capacitance and RH within the range from 20 to 99.9%. Measurements were taken for the Al/DNA/Al humidity sensor for non-radiated samples (2, 4, 6 and 8 min of irradiation) at 0.8 KHz and 1 V^29^,^30^,^31^. From the experimental results, the capacitance was observed to increase with higher humidity, which demonstrates sensitivity to humidity in the studied range. Higher water molecule content at high humidity levels increases dielectric permittivity constant, thereby acting to improve the capacitance of the device^18^,^32^. This in turn increases conductivity as a result of the rise in electron transport along the dsDNA helix^33^. In the case of decreasing humidity, a deviation was observed instead. Table 1 shows the capacitance values in three distinct parts. In the range 99.9%, the highest capacitance values were 92.736, 62.103 and 55.102 nF for 2 min, 6 min and in non-radiated samples, respectively. For RH of 45%, 2 min registers the highest value (3.1506 nF) followed by 8 min (0.44304 nF) and 4 min (0.30054 nF). This trend changes again at RH value of 20%, where 2 min is the highest followed by 6 and 8 min (0.46463, 0.14945 and 0.1425 nF, respectively). In the last two cases, the non-radiated samples registered the lowest capacitance values contrary to the highest RH environment.

At high humidity levels, water molecules play an important role towards improvement in conductivity in which case, the increase in electron transfer along the dsDNA helix may lead to the improvement in charge conduction. The charge transfer phenomena may therefore play an important role in the sensing mechanism, which could have primarily lead to the insignificant base-line shift and faster recovery time. This change in the electrical properties of the Al/DNA/Al structure due to the change in RH may therefore be generally attributed to the absorption of water molecules and the increase of the number of holes due to tracks made by the alpha particles upon bombardment.

In is known that alpha particles lead to primary and secondary ionizations in atoms upon interaction. These processes in turn produce several types of ions and excited molecules in cells ultimately causing intermolecular bond biomolecule cleavage. Cleavage occurs within the cytoplasm and other intracellular components besides the cell nucleus. As such, DNA molecule being double stranded exhibits cleavage in one or both the strands. The DNA molecule has characteristic capability to repair single-strand damage; but a scission in double-stranded DNA repair to its original form is not trivial. However repair of double-stranded scission of the DNA molecule may cause gene mutations as a result of the exposure to radiation^34^.

Fig. 1(b) shows the relationship between capacitance-RH within the range of 20 to 99.9%. The non-radiated samples showed a significant increase with increasing humidity with a maximum at 75%. After which, the value begins to drop dramatically and the irradiated samples now register higher capacitance values. The sample irradiated for 2 min (92.736 nF) registers higher value compared to 6 (62.103 nF) and 4 min (33.549 nF).

**Figure 2:**
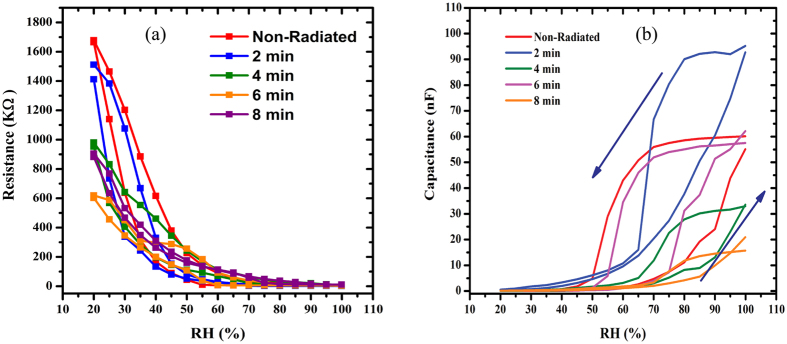
Relation between the capacitance and resistance with humidity for the Al/DNA/Al junctions.

The relationship between humidity and the resistance within the range from 20 to 99.9% RH is demonstrated in Fig. 2(a). Here the resistance decreases with increasing humidity, which clearly depicts the sensitivity to humidity in the studied range. Generally, the highest value was observed in the non-radiated samples in the 20 to 45% RH range, followed by samples irradiated for 2 and 4 min. In the case of decreasing RH environment (99.9–20%) the non-radiated samples still record the highest values until 30% RH. However, samples radiated for 8 min registers a higher resistance value in the 35–99.9% range. All other irradiated samples demonstrate higher values compared to the non-radiated samples in the 50–75% range. Fig. 2 demonstrates the trend for the resistance with RH, which shows a clear variation between the range 45–75% RH. The trend in the resistance and capacitance can also be observed clearly above 45% RH as shown in Table 2. The readout of the resistance values obtained from the device in response to the RH values was also achieved (Fig. 2(a)), which demonstrated the exponential decrease in the resistance values relative to the increasing RH. The ionic dissociation of the water molecules may have led to this phenomenon that acts to increase the film conductivity. Capacitance values meanwhile initially increased gradually within the range 20–40% RH, followed by rapid rise within 45–99.9% range as shown in Fig. 2(b) depending on irradiation time (Table 1).

As discussed, the resistance in general decreased exponentially (Fig. 2(a)), while the capacitance increased following a S-shape (Fig. 2(b)). This could be attributed to the charge transfer mechanism between the DNA and the water molecules. The observed changes in the mechanism results from the decrease in resistance in response to increase in H_2_O molecule concentration and displacement currents, and the concentration of charge carriers doped by water molecules^35^, which causes an exponential variation of conductance of the DFC nanotube networks with the RH. This is in contrast to observation by Paul *et al.*, who in their work indicated that the reason for the low output signal current from the networks may be due to it being unsuitable for use in low RH sensors. Since the output signal current of the detector rises exponentially with increase in RH, the device display elevated sensitivity especially at higher RH^20^.

**Figure 3:**
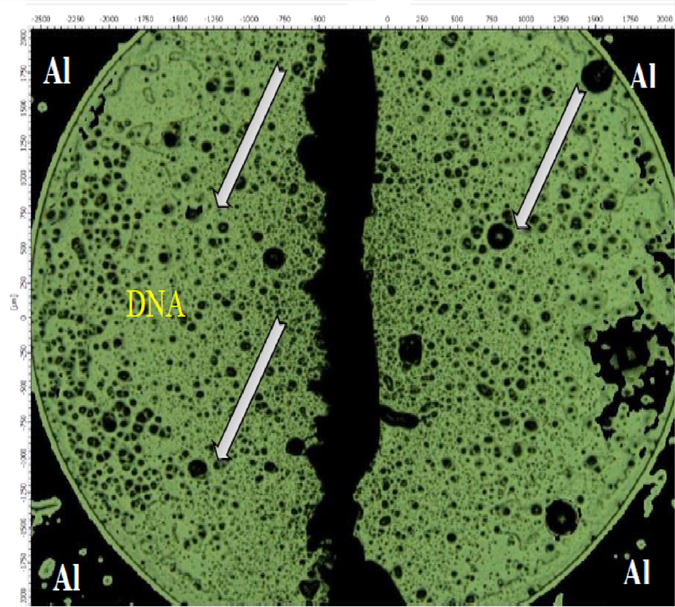
Optical microscope (Infinite Focus, Alcona, Austria) image showing the number of alpha particle tracks on the Al/DNA/Al sensor after irradiation.

The resistance meanwhile decreased exponentially and was attributed to adsorption of water molecules by the DNA. This change is explained as follows; DNA consists of three portions; bases, sugars, and phosphoric acids. The hydrophilic phosphoric acid around the base pairs of DNA caused the water molecules to be easily absorbed and form hydrogen bonds between the phosphoric acid and the water molecule. Resistance values quickly decline as the RH increases and tend to saturate for humidity above 99.9%. These phenomena agree with ionic conduction at higher humidity scale^36^. The decline of resistance with increasing humidity meanwhile displays sensitivity to humidity within the studied range. Electronic instrument features are significantly affected by absorption of water molecules on the surface of the DNA film being the active layer. Water molecules absorbed on the surface of the Al/DNA/Al film have great dipole momentum, which leads to the increase of the charge carrier density^37^. Number of alpha particle tracks acting as micro-scale pores as shown in Fig. 3 also plays a vital role in the charge conduction upon easy absorption of water molecules, which in turn decreases the resistance of the device.

Otsuka *et al.* studied the humidity influence on the electrical conductivity of DNA thin film^13^. They observed that the ionic conduction was overcome by the electrical conduction. The capacitance values increasing with the increase in RH% indicated the proton transfer through the physically adsorbed layer of water, which improved with increasing humidity. At higher RH, the proton conductivity dominates the ionic conduction. As RH increases, intermolecular conductivity is increased rapidly mainly due to the increase of local dielectric constant^14^. In the latter case, the exponential dependence of the conductivity was attributed to the adsorption of water molecules on the nanostructured film surfaces. As a result of the current increasing with humidity, it is believed that the charge carriers are the H^1^ and OH^−^ species produced by water adsorption. The ions separate and recombine according to the Grotthus mechanism, which describes the passing of protons through the cooperation of neighboring water molecules^38^.

**Figure 4:**
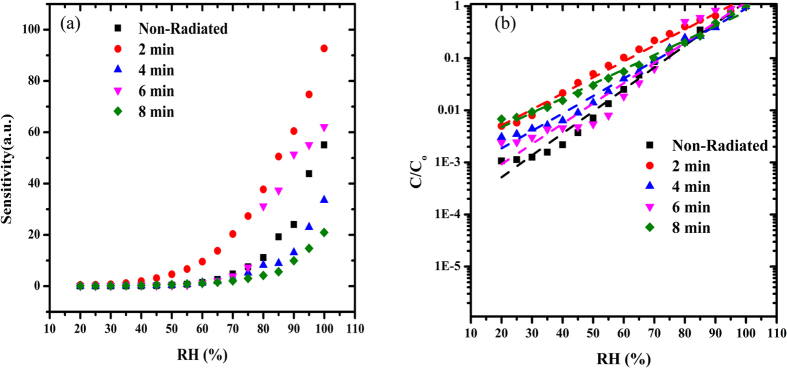
Graph (**a**) demonstrates the sensor’s sensitivity and (**b**) shows the results for the capacitance- humidity relationship of the Al/DNA/Al humidity sensor.

Fig. 4(a) demonstrates the sensitivity of the pre and post-radiated Al/DNA/Al junctions in the RH range of 20–99.9%. The sensitivity showed an exponential behavior upon increase in the RH value. Highest value was observed at 2 min, while significant increase in sensitivity was observed for 6 min under 75% RH. The humidity detecting capabilities of this type of capacitive device rely on factors such as the gap between the electrodes and the area of the electrodes^2^. Al/DNA/Al device can be considered as a surface plate capacitor, assuming that the face edges of the electrodes act as parallel layers. As such, the capacitance of the sensor can be measured using the following method^10^,^39^,^40^.


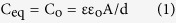


where C_o_ is the initial capacitance, ε represents the relative dielectric constant, ε_o_ the absolute permittivity, A is equal to the area of the surface and d the distance between the electrodes. Eq. (2) meanwhile expresses the capacitance influenced by the humidity effect. This equation can also be obtained by fitting the sensor under higher humidity levels;


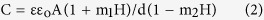


where H is the relative humidity level and m_1_ and m_2_ are the constants. In the case of relative capacitance, Eq. (2) can be rewritten as;


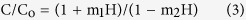


From Eq. (3), the following simple equation can be derived;


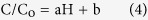


Fig. 4(b) shows the experimental and simulated results where the latter results were calculated using Eq. (4). Table 2 lists the sensitivity and value a of the slope in Fig. 4(b) and the value b from Eq. (4) for both the non-radiated and radiated structures (2, 4, 6 and 8 min).

**Figure 5:**
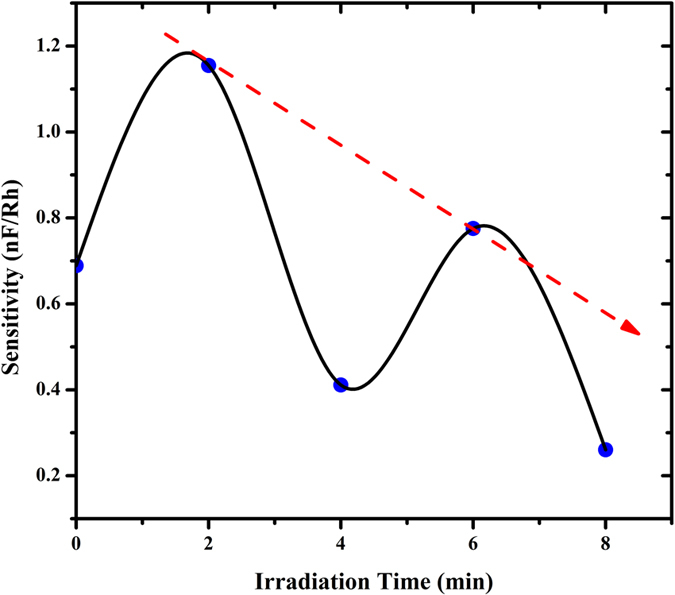
Graph demonstrates the hypersensitivity phenomena of DNA at four minutes of irradiation time. The dashed line illustrates the trend normally observed for other materials when there is no occurrence of the hypersensitivity phenomena as shown in a similar work with survival curve fraction with irradiation dose by Munetoshi *et al.*^41^.

Fig. 5 demonstrates the relationship between sensitivity and irradiation time. It shows that sensitivity fluctuates with alpha radiation time, possibly due to the occurrence of the hypersensitivity phenomena indicated by the drop in sensitivity at four minutes. At this point, the DNA seeks to self-protect itself against the radiation and humidity effects as depicted by the survival curve phenomena demonstrated by Hassan *et al.*^41^ and others^42^,^43^,^44^.

Sensitivity is calculated using the following relation;

Sensitivity = Capacitance at high humidity (99.9%) − Capacitance at low humidity (20%)/(high humidity - low humidity) as shown in Eq. (5).


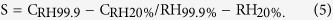


**Figure 6:**
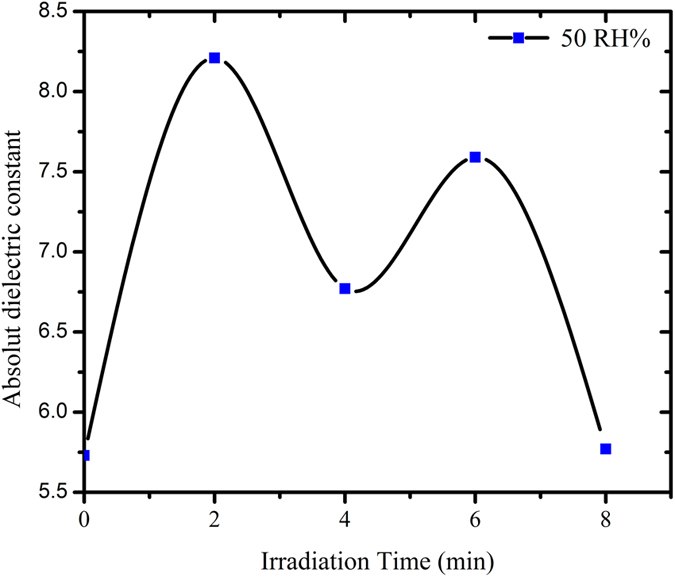
Variations in the dielectric constant of DNA with irradiation time.

The dielectric constant from the fits made from Eq. 4 was calculated and tabulated in Table 3. Values for the dielectric constant were calculated at RH = 50% for all the samples^36^, which was observed to peak at irradiation times of 2 and 6 min. It is expected that upon initial exposure to alpha particles, a deviation from the general dielectric constant value (in this case, about 5.73) could be observed. A maximum value of 8.21 is calculated at irradiation time of 2 min, which indicates the onset of DNA damage. It is understood that tracks are formed upon bombardment of alpha particles. These tracks involve an increase in the number of charges due to the linear energy transfer (LET) from the alpha particle^34^,^45^,^46^. As a result of a large number of material excitation by these particles along its path, an increase in the number of charges can be expected. This in then increases the dielectrical constant as shown at 2 min of irradiation. Further irradiation however may initiate the self-repair mechanism within the DNA structure, effectively undergoing recombination. This is calculated as a decrease in the dielectric constant at 4 min of irradiation. These process is continued accordingly with further irradiation until 8 min, where the dielectric constant becomes almost the same as with the non-radiated DNA (Fig. (6)).

**Figure 7:**
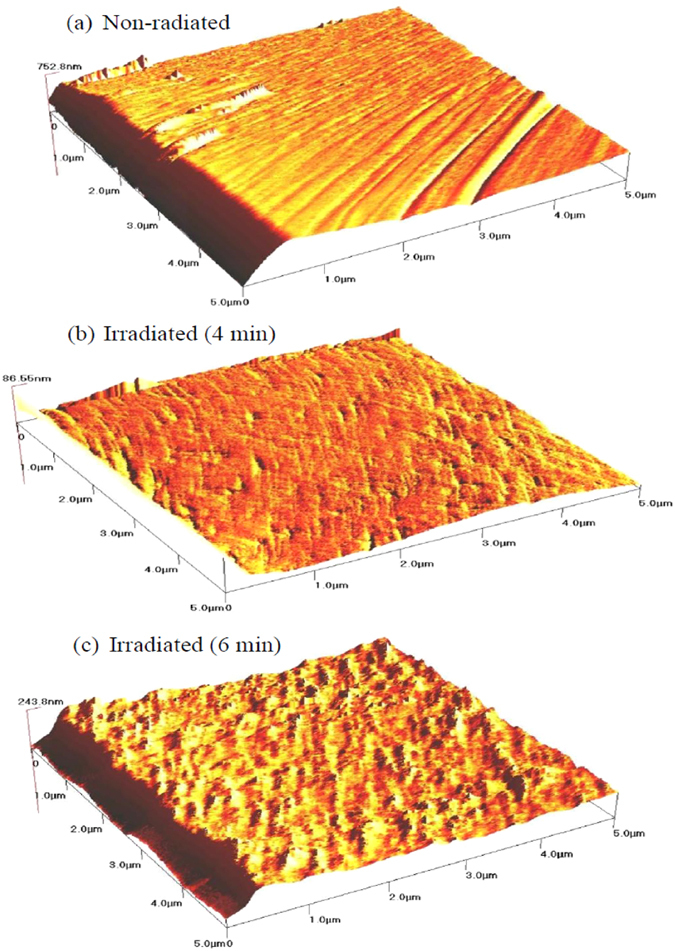
AFM images of (**a**) non-radiated and (**b,c**) radiated Al/DNA/Al sensors.

The surface morphology of the Al/DNA/Al thin films was examined using Atomic Force Microscope (AFM) (Q-Scope Series, Ambios Technology, Germany) and shown in Fig. 7. The “spongy” looking film surface consists of water molecules due to absorption and numerous tracks resulting from the alpha particle irradiation. This also ensures efficient distribution of the water molecules and therefore increases electrical response to the humidity. To evaluate the performance of the Al/DNA/Al sensor, it’s response and recovery behavior can be examined experimentally, which is considered one of the most significant features for evaluating any type of sensors^10^,^30^,^47^.

**Figure 8:**
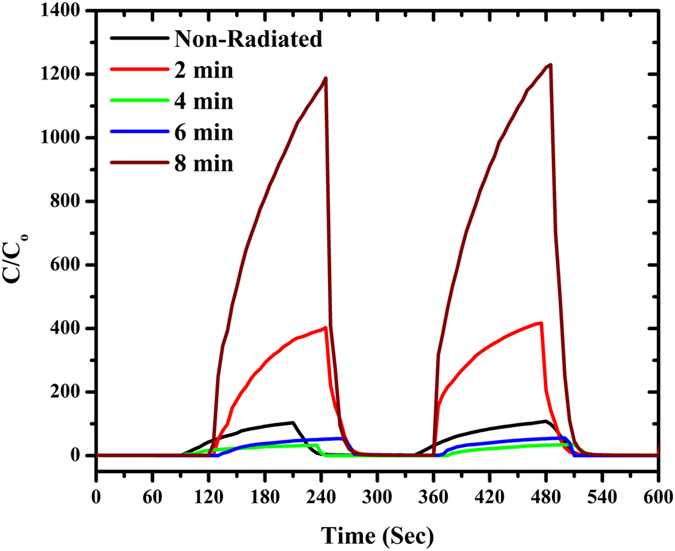
Response-recovery graph for the Al/DNA/Al humidity sensor.

The profiles in Fig. 8 demonstrate the capacitive response of the Al/DNA/Al structure exposed to a fast variation of humidity (5–95% RH), which was observed to be rapid. The electrical response of the sensor becomes unstable when exposed to a RH value of 95%, followed by a rapid and sharp change back to its unique values within 5 s upon replacing the tested vapor condition. Response and recovery time characteristics of the Al/DNA/Al sensor were measured at a frequency of 800 Hz under a RH condition of 5%. According to the graphs in Fig. 8, the sensor response time (humidification from 5% to 95% RH) was 26 s. The relative capacitance of the sensor increased from 4.0 (5% RH) to 1188 nF (95% RH). When exposed to the maximum humidity of 95% RH, values observed were 106, 420, 34, 55 and 1188 nF for the non-radiated, 2, 4, 6 and 8 min, respectively, while the recovery time (drying effect, 95 to 5% RH) was 78 s. Both types of responses (humidification and drying) illustrates obvious changes due to some hysteresis effect^48^. These changes are highly dependent on the thickness of the structure^49^ and may also be attributed to permanent structural defect due to the alpha irradiation.

The electrical characteristics of the Schottky barrier height connections are very sensitive to the features of the metal-semiconductor (MS) interface. As such, the I–V characteristics of the connection are suitable displays of the interface features. For a Schottky barrier diode, the thermionic emission theory predicts that the I–V characteristics at forward bias V are given by^50^;


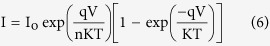



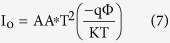


where A^^*^^ = 120 Am^−2^K^−2^ is the effective Richardson constant for Al^51^, A is the diode area, K is the Boltzmann constant, q is the electronic charge, T is the temperature, n is the ideality factor and ф_b_ is the barrier height (in eV). For values of V > 3 KT/q, the ideality factor can be obtained from Eq. (6), be re-written as;


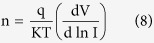


**Figure 9:**
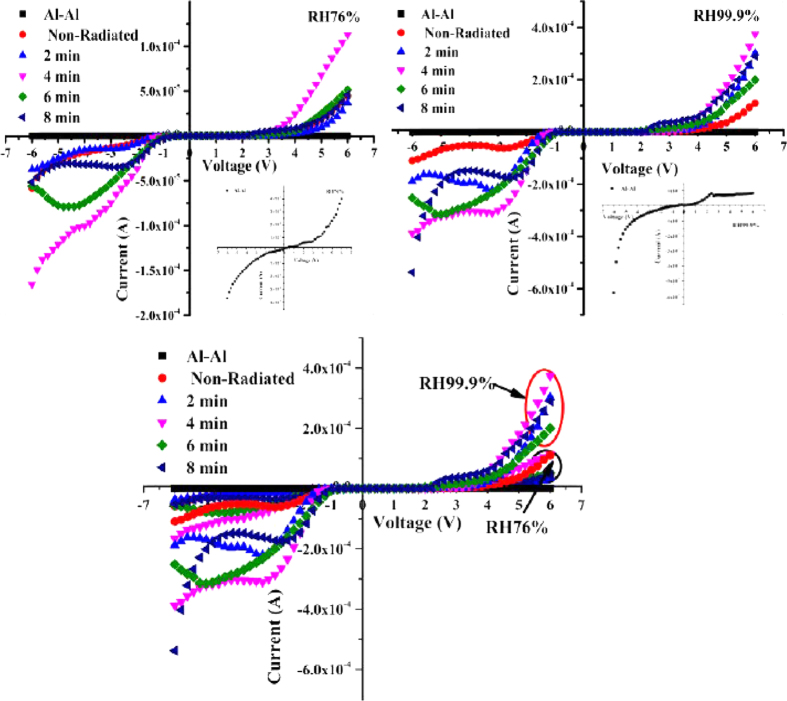
Graphs demonstrate the relationship between current and voltage under different humidity conditions.

Fig. 9 provide the I–V characteristics of the fabricated Al–Al and Al/DNA/Al Schottky barrier diodes under normal (a) and high (b) humidity conditions. The profiles demonstrate highly rectifying behaviors with the presence of the DNA as observed from all the profiles. Al–Al structures clearly show an ohmic behavior further confirming the rectifying nature of the DNA layer.

**Figure 10:**
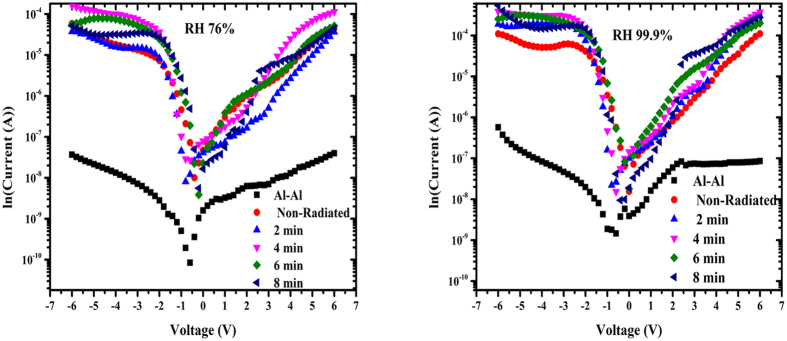
Profiles demonstrate the relation between ln I–V under irradiation effect and humidity condition.

**Figure 11:**
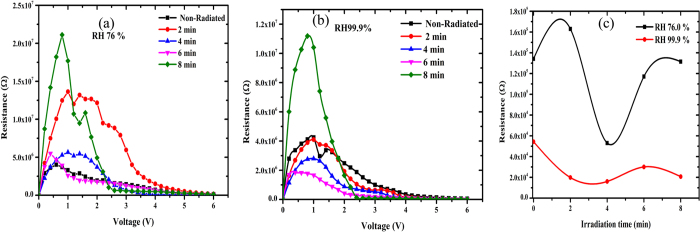
Profiles in (**a**) shows the relationship between the resistance and the voltage at 76% RH, (**b**) demonstrates the behavior of resistance against voltage at 99.9% RH and (**c**) the relation between the series resistance and irradiation time for 76 and 99.9% RH.

**Figure 12:**
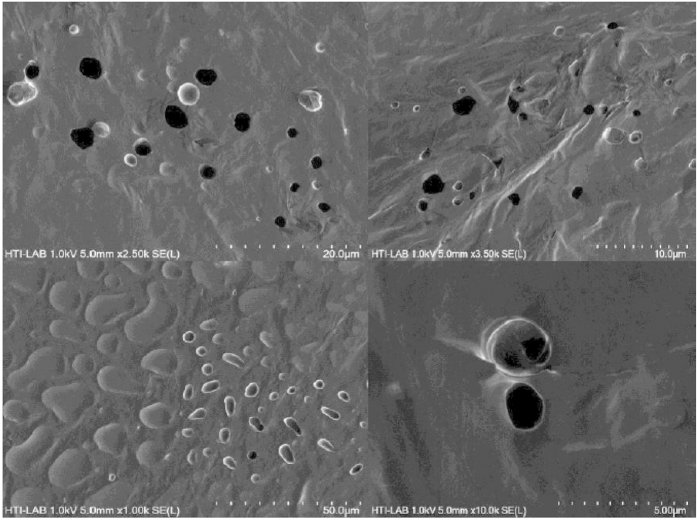
The alpha particle tracks on DNA film irradiated for 2 min.

The ideality factor determined from the slope of the linear region of the forward bias (ln (I)–V) characteristic through the relation in Eq. (8) is a measure of conformity of diode to pure thermionic emission^52^,^53^. The ideality factor of the Al/DNA/Al junctions was larger than unity in the present work. An ideality factor greater than unity is generally attributed to the presence of a bias dependent distribution of low Schottky barrier height (SBH) patches (or barrier inhomogeneity), re-arrangement of electrons and holes in the depletion regions, and bias dependence of voltage of SBH^54^, the thickness of organic film^55^, series resistance^56^ and temperature^57^. Fig. 10 demonstrates the fact that DNA is the sensing element and not the Al metal. The ideality factor shows fluctuations of Al/DNA/Al based junctions fabricated in this work under the humidity condition calculated using Eq. (8). Fig 11 and 12 represent the forward and reverse I–V characteristics of the Al/DNA/Al Schottky diode before and after alpha irradiation under different humidity conditions (76, 99.9% RH). It is found that both forward and reverse current generally increases after the irradiation. The results of Fig. 9 agree with another work by Jo *et al.* obtained under different humidity conditions. The highly rectifying curve and inverted S shape in this work at high relative humidity are clearly attributed to water molecules adsorbed by the DNA. Dielectric constant increase lead to the increase of the ionic conduction^58^ which enhances the dissociation of water to H^+^ and OH^−^ and the different mobility between H^+^ and OH^14^. The formation of electrical double layer through the redistribution of ions at the contacts between DNA molecules and electrodes leads to the highly nonlinear behavior seen on the I–V plots. Increasing RH also acts to increase the hysteresis^14^, which can be attributed to the increase in reverse current with dose as a result of generation of carriers in the bulk depletion region. Lattice defects are also induced due to the reverse current, which is proportional to the concentration of minority carriers near the junction.

The values of series resistance are calculated from the junction resistance formula R_S_ = 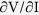 from the I–V features of the diode. The series resistance (R_S_) versus voltage of the surface type Schottky diode is demonstrated in Fig. 11. From the figure, it can be concluded that at low voltages (≤2.0 V), R_S_ values were the highest for 8, 2 and 6 min, respectively in reducing order, followed by the sample radiated for 4 min and non-radiated at 76% RH. At 99.9% RH meanwhile, the series resistance values were the highest for 8 min, non-radiated and 2 min in reducing order followed by the sample radiated for 4 and 6 min. However above 2.0 V, the series resistance values become insignificant.

From Fig. 11(a,b), the shunt resistance becomes the highest at 1 V for all the samples and under the humidity condition. However, from Fig. 11(c), the series resistance at 99.9% RH reduces compared to at 76% RH due to the increase in H_2_O molecules (Table 4).

According to Ha *et al.*, the electronics properties of DNA such as the I–V profiles are generally influenced by the contact, bulk (DNA channel) and intermolecular features under humidity effect^14^. It could be also assumed that the majority of response is initiated by the the Schottky metal (Al)-semiconductor (DNA) contact.

Fig. 12 shows the tracks of alpha particles on the DNA film irradiated for 2 min using a Field Emission Scanning Electron Microscope (FESEM). The tracks seen in the image are similar to pores, agreeing well with the honeycomb structure that has been demonstrated for its’ potential applications in electronic, optical and micromechanical devices^59^,^60^.

## Conclusions

Humidity sensing properties of the DNA films deposited by drop casting method were investigated. Electrical characterization studies were carried-out using I–V profiles under humidity fluctuations and Schottky diode parameters such as ideality factor (n), barrier height (ф) and series resistance (R_S_) were extracted. The results generally indicate an increase in the ideality factor with increasing humidity, while the series resistance and the barrier height values register attenuations with increase in the humidity. The influence of RH on capacitance and resistance of the sensor was studied in the humidity range of 20 to 99.9% RH. The sensors fabricated in this work demonstrated higher sensitivity across all the humidity range tested. Results also indicated that with the rise in humidity, capacitance increases while the device resistance dropped. Resistance and capacitance-humidity relationships recorded significant variations in the range of 50–99.9% and 45–75% RH. The resistance of the film generally reduced from a high of 1678 to a low of 6.523 KΩ and 1512 to 1.801 KΩ for the non-radiated and 2 min of alpha irradiation, respectively. The series resistance value increased after the irradiation for 2 min, followed by a decrease at 4 min (≈53.0755 KΩ) for characteristic to the hypersensitivity phenomena, which may correspond directly to the damage and repair mechanism in DNA, It was also shown that under the effect of increasing humidity, the capacitance of DNA thin film increased from 0.05894 to 92.736 nF for both the non-radiated and irradiated samples. These observations show the exciting possibility of utilizing Al/DNA/Al Schottky barriers as potentially sensitive humidity sensors and may even provide a platform to allow the study of charge transfer mechanism in DNA damage and repair processes.

## Materials and Methods

### Preparation of DNA solution

A simple preparation procedure of mushroom DNA extracted from colonies of fruiting bodies was used for Polymerase Chain Reaction (PCR) amplification. The procedure starts with the collection of minute quantities of mycelium (0.1–1.0 g) from a colony of the fruiting body (Stipe) of a mushroom species using a sterilized tweezer. Standard procedures according to Hibbett *et al.*^61^ were further employed to yield pure DNA samples prior to the PCR process. The DNA of all samples was amplified by PCR (PTC–100 TM, MJ Research Inc., Ramsey, MN, USA) using universal primers ITS1 forward (5′-TCC GTA GGTGA AC CTGCGG-3′) and ITS4 reverse (5′-TCCTCCGCTT ATT GATATGC-3′). Amplification reactions were performed in a total volume of 50.0 μl containing 10× PCR buffer 4.0 μl, dNTP mix, 2.5 μl of each primer, 1.0 μl of Taq polymerase (Cosmo, Seongnam-si, Gyeonggi-do, Korea), 4.0 μl of genomic (Template DNA), and 26.0 μl of sterilized distilled water. PCR amplification was carried-out in 30 cycles at 94 °C for 30 min and denatured at 50 °C for 60 min, followed by annealing at 72 °C for an extension of 1 min. Initial denaturing at 95 °C was extended to 5 min and the final extension was at 72 °C for 5 min^62^,^63^.

### Fabrication of the Al/DNA/Al sensor

**Figure 13:**
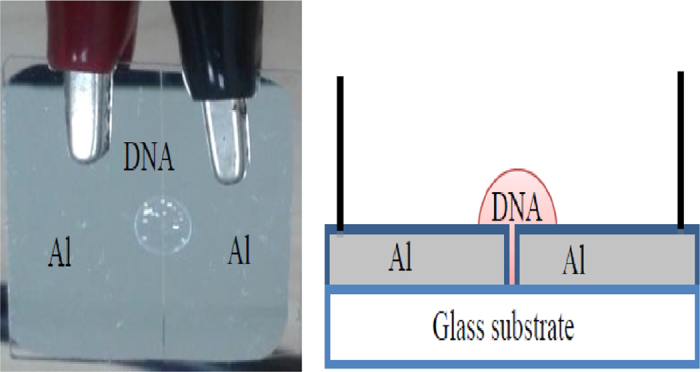
Image (left) and schematic diagram (right) of the Al/DNA/Al humidity sensor.

A glass substrate was cleaned for 15 min using deionized water (18.2 MΩ.cm, Barnstead Nanopure II water system, Lake Balboa, CA, USA) in an ultrasonic cleaner and later dried in a dust-free environment. Thin films of Al (thickness ~325 nm) were deposited on the glass substrate using an Edward Auto 306 vacuum coater with a diffusion pumping system (Edward Auto 306, West Sussex, United Kingdom) and Al metal wire (Kurt J. Lesker, Hudson Valley, PA, USA) of 99.999% purity). While depositing the Al thin film, the pressure inside the chamber was kept at 10^−5^ mbar, whereas the deposition rate was maintained at 0.1 nm/s and the gap length and width of gaps (between the electrodes) was 25 mm and 400 μm, respectively. After which, formation of the organic DNA layer was carried-out by using a micro-syringe (Hamilton micro syringe, 10 μl) containing the pre-prepared DNA solution (concentration of 1.80 ng/μl). The fabricated device was then kept in a 1 K Cleanroom to allow self-assembly overnight. Sample irradiation by alpha particles was achieved using a ^241^Am source with an activity of 150 nCurie and t_1/2_ of 457 years for periods of 2, 4, 6 and 8 min. The top and cross-sectional view of the Al/DNA/Al surface-type Schottky diode fabricated is shown in Fig. 13.

**Figure 14:**
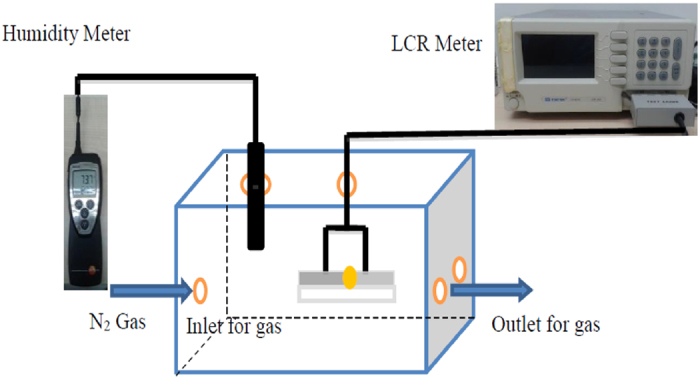
The experimental setup used in the work using LCR and humidity meters (photo credit by Hassan Maktuff Jaber Al-Ta’ii) from the Low Dimensional Materials Research Centre (LDMRC), Department of Physics, University of Malaya.

The humidity meter and the Al/DNA/Al placed in a closed chamber were exposed to irradiation of alpha particles in a controlled humidity environment. The chamber has built-in input and output valves for gas flow. Nitrogen gas was then passed through water and then channeled into the chamber to control and maintain a certain humidity level within the chamber. LCR Meter (Instruments Instek LCR-829 LCR Meter) was used to measure the capacitance of the sensor. The *in-situ* capacitance and resistance values versus RH measurements of the Al/DNA/Al sensor at ambient temperature (25 ± 1 °C), were carried-out by placing the device in the hermetically sealed humidity chamber capable of providing a humidity range of 20–99.9% RH. Fig. 14 illustrates the experimental setup used for the measurements. Direct I–V measurements were carried-out under relative humidity for 76% and 99.9% using a Keithley 236 Source Measurement Unit (SMU).

## Additional Information

**How to cite this article**: Al-Ta’ii, H. M. J. *et al.* Humidity influenced capacitance and resistance of an Al/DNA/Al Schottky diode irradiated by alpha particles. *Sci. Rep.* **6**, 25519; doi: 10.1038/srep25519 (2016).

## Figures and Tables

**Table 1:** Capacitance and resistance values registered under different relative humidity.

Irradiation Time (min)	Capacitance (nF)			Resistance (KΩ)		
	20%	45%	99.9%	20%	45%	99.9%
Non-radiated	0.05894	0.20463	55.102	1678	379.4	6.526
2	0.46463	3.1506	92.736	1512	153.3	1.801
4	0.103	0.30054	33.549	980.3	344.4	9.194
6	0.14945	0.29634	62.103	619.6	287.5	4.017
8	0.1425	0.44304	20.938	905.2	235.1	11.1

**Table 2:** Sensitivity values and other parameters measured for the Al/DNA/Al Schottky barrier diode type humidity sensor.

	Non-radiated	2 min	4 min	6 min	8 min
Slope (a)	0.02773	0.04214	0.03356	0.03889	0.03068
b	−2.8779	−4.12762	−3.40147	−3.81196	−2.90167
Adj. R-Square	0.99023	0.99199	0.98588	0.93011	0.98775
Sensitivity	0.6889	1.15484	0.4112	0.77539	0.26027

**Table 3:** Dielectric constant for DNA exposed to different irradiation time.

Irradiation time (min)	Slope (a.u)	Absolute dielectric constant	Log (dielectric constant)
0	0.02771	5.73	0.75800
2	0.04214	8.21	0.914
4	0.03356	6.77	0.8305
6	0.03889	7.59	0.8799
8	0.03068	5.77	0.76

**Table 4:** The values of the Schottky diode parameters.

Irradiation Time (min)	n		Ф (eV)		R_s_ (KΩ)	
	RH 76%	RH 99.9%	RH 76%	RH 99.9%	RH 76%	RH 99.9%
Non-Radiated	1.04102	1.09508	0.5809	0.5807	134.2362	54.33274
2	1.0005	1.1694	0.7142	0.6469	162.9461	19.8621
4	1.0291	1.30604	0.6327	0.6026	53.0755	16.0481
6	1.12012	1.23286	0.6084	0.5855	117.12803	30.03404
8	1.8923	1.25231	0.7284	0.6308	131.4886	20.6821
